# The inner workings of a miniature eye

**DOI:** 10.7554/eLife.105736

**Published:** 2025-02-05

**Authors:** Gregor Belušič

**Affiliations:** 1 Department of Biology, Biotechnical Faculty, University of Ljubljana Ljubljana Slovenia

**Keywords:** *Megaphragma viggianii*, vision, visual system, miniaturization, compound eye, Other

## Abstract

The first complete 3D reconstruction of the compound eye of a minute wasp species sheds light on the nuts and bolts of size reduction.

**Related research article** Makarova AA, Chua NJ, Diakova AV, Desyatirkina IA, Gunn P, Pang S, Xu CS, Hess H, Chklovskii DB, Polilov AA. 2025. The first complete 3D reconstruction and morphofunctional mapping of an insect eye. *eLife* **14**:RP103247. doi: 10.7554/eLife.103247.

How small can an organ get while remaining functional? Parasitic wasps from the *Megaphragma* genus are ideal organisms in which to investigate this question. Using a range of senses, including vision, they locate minuscule insects, known as thrips, inside which they lay their eggs (Polaszek et al., 2022). Upon hatching, the wasp larvae eat the insides of their hosts, pupate and emerge as adults from the remains, ready to repeat the cycle. This parasitic lifestyle imposes severe selective pressures; in particular, much like matryoshka dolls, the parasites generally need to be smaller than the animals they take advantage of. Since thrips are less than a millimeter long, *Megaphragma* wasps are minuscule, often barely reaching a fifth of a millimeter. Such extreme miniaturization requires drastic space-saving adaptations; the brain of *Megaphragma viggianii* wasps, for example, has fewer neurons which also lack nuclei (Polilov, 2012). Still, this reduction process can only go so far – especially for structures like the eye, which have strict size limitations due to the laws of physics and the nature of light.

Insects possess compound eyes formed of discrete, elongated units known as ommatidia. Like pixels in a camera sensor, each ommatidium samples a portion of the visual space and provides a fraction of the overall perceived image. To do so, ommatidia feature a lens that focuses and directs light onto the rhabdom, a structure stemming from photosensitive cells that convert light packets (photons) into electrical signals which are then relayed to brain neurons. To function properly, these building blocks must meet certain size requirements: in particular, the lenses must be large enough to focus an adequate amount of light from a certain direction without distortion, and the rhabdom must be sufficiently thick to guide the incoming photons.

With only 29 ommatidia, the eyes of *M. viggianii* wasps are one the smallest amongst animals that exhibit complex behaviors and spatial navigation (Figure 1A). Previous work has started to shed light on how their visual system can accommodate such extreme miniaturization (Chua et al., 2023). Now, in eLife, Alexey Polilov and colleagues – including Anastasia Makarova as first author – report the first complete 3D map of the compound eye of adult *M. viggianii* wasps with unprecedented detail (Makarova et al., 2025).

**Figure 1.**
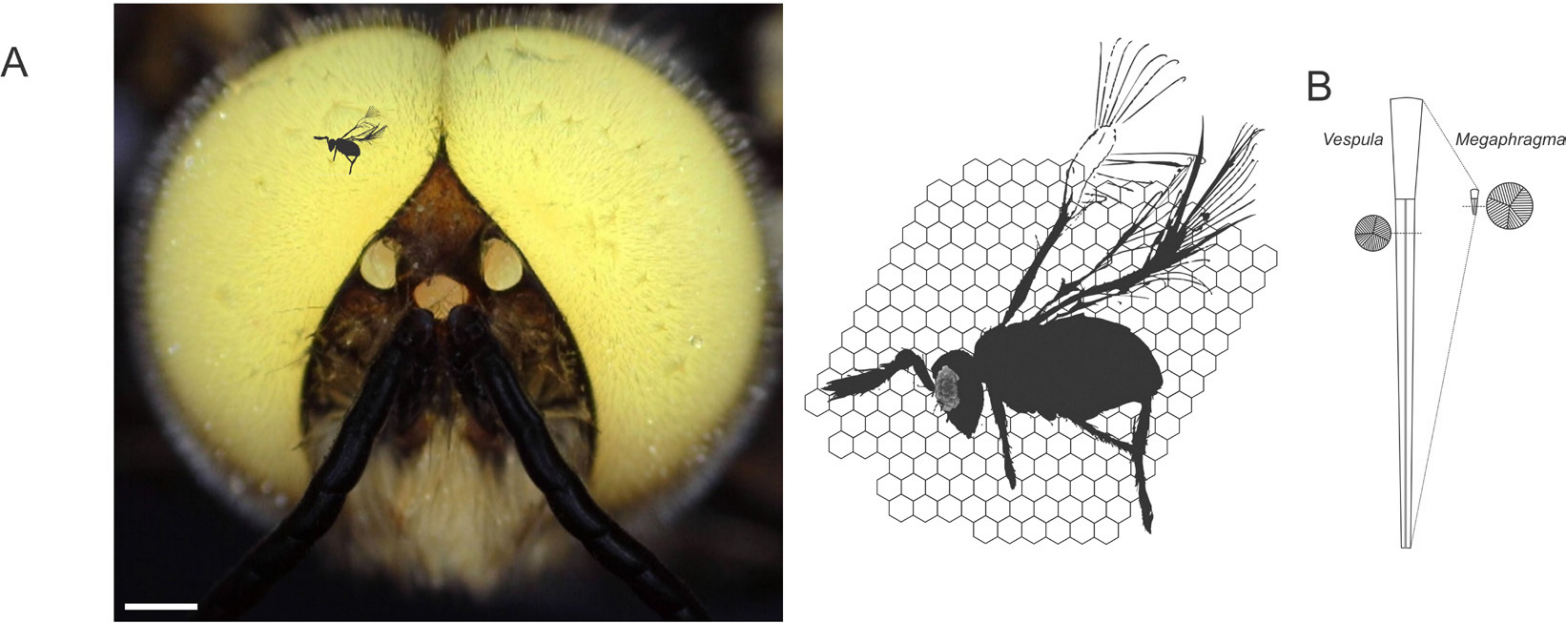
Size scaling of hymenopteran eyes, from *Megaphragma viggianii* to common wasps and honeybee drones. (**A**) *M. viggianii* (black silhouette), honeybees (head of an albino drone with white eyes; left) and other bee-sized insects such as the common wasp (*Vespula vulgaris*) all rely on their vision to navigate their environment. Despite striking size differences, these species possess similar compound eyes consisting of individual units, or ommatidia (right; ommatidial facets of *V. vulgaris* represented as hexagonal shapes behind the full body of *M. viggianii*, whose ommatidia are depicted as small circular shapes in the eye). Scale bar, 0.5 mm. (**B**) The work by Makarova et al. sheds light on the organization of the eye in *M. viggianii* wasps. As in most insects, ommatidia in *V. vulgaris* (left) and *M. viggianii* (right) wasps are shaped like slightly tapered columns with a lens near their surface (top), and photosensitive structures (known as rhabdom; circular insets) further down inside. In *M. viggianii,* each ommatidium is only 20 µm long (compared to 350 µm in *V. vulgaris*), and the diameter of its lens reaches around 8 µm (25 µm in *V. vulgaris*). Yet the width of the rhabdom remains similar between the two species (1.5 µm diameter in *V. vulgaris* vs. 2 µm in *M. viggianii*).

Image credit: Honeybee drone picture by Gregor Belušič (CC BY 4.0); *M. viggianii* silhouette adapted from Figure 1B of Polilov, 2017 (CC BY 4.0); ommatidia silhouette adapted from Figure 4A–B of Gutiérrez et al., 2024 (CC BY 4.0).

The team, which is based at Lomonosov Moscow State University and various institutions in the United States, exploited one of the advantages conferred by the small size of the insect; namely, that its entire body could be sectioned and imaged via electron microscopy at once (Polilov, 2017). Imaging revealed that the eye is formed of 478 cells, with a single ommatidium bringing together nine photosensitive cells, four cone cells (that focus light), and two primary pigment cells containing granules full of opaque compounds.

Further analyses showed that the diameter of the lens in an ommatidium is only 8 µm at most, which is hardly sufficient to focus light onto photoreceptors without distortion. However, the underlying rhabdom has retained a relatively broad cross-section, 2 µm, which is similar to that found in large diurnal insects (Figure 1B). Together, the lens and rhabdom are therefore able to efficiently capture enough light for the eyes to perform during the day. In addition, the sides of the ommatidia are coated with very dense layers of pigment granules that optically isolate the rhabdoms, blocking stray light and therefore contributing to image formation.

Examining cell structure revealed that, unlike neurons in the brain of *M. viggianii*, eye cells have nuclei; probably due to optical constraints, these cells remained large enough to escape extreme miniaturization and retain an essential organelle lost elsewhere in the visual pathway. Finally, Makarova et al. show that photoreceptor cells are packed with mitochondria, suggesting that vision is metabolically costly to the wasps.

In many insects, an eye region known as the dorsal rim area is part of a visual system used to analyze polarized light from the sky, which serves as a stable spatial reference when animals travel in arbitrary directions or across longer distances (Labhart and Meyer, 1999). Surprisingly, in *M. viggiani* wasps, about a third of the available ommatidia, located dorsally, show specializations for the detection of polarized light; this includes a special rhabdom geometry, smaller optical parts and even cell nuclei inserted into the optical pathway that may play an optical role, similar to eye structures facilitating polarized light navigation in honeybees (Meyer and Labhart, 1981). The compound eye thus shows a remarkable resource allocation, with ~30% dedicated to navigation and ~60% to image formation. Lastly, the analysis revealed the presence of three photoreceptors disconnected from the eye optics behind the first ommatidia in the dorsal margin; these structures possibly contribute to other biological processes requiring light detection, such as the regulation of circadian rhythms.

Overall, the work by Makarova et al. represents a valuable quantitative morphological reference for the future studies of compound eyes and miniaturized organs in visual physiology and cell biology. It will inform the design of miniaturized imaging sensors, enable the numerical modelling of small compound eyes, and support the framework for the understanding of cellular architecture in eukaryotic organisms.
